# Phytosociology and biodiversity of roadside herbs in a salinity-affected coastal area of Bangladesh

**DOI:** 10.1016/j.heliyon.2021.e07813

**Published:** 2021-08-17

**Authors:** Abu Selim, Ehsanul Bari, Md. Hasibur Rahaman, Mohammad Mahfuzur Rahman

**Affiliations:** Department of Environmental Science and Technology, Jashore University of Science and Technology, Jashore, 7408, Bangladesh

**Keywords:** Plant biodiversity, Herbaceous plant, Coastal area, Phytosociology, Soil salinity

## Abstract

Soil salinity in the coastal areas of Bangladesh has been rising. The responses of forest communities to the rise of salinity are somehow documented. However, the adjustments of non-forest plant communities against salinity are still limited in the literature. This study explains the phytosociology and the herbaceous plant biodiversity along with the soil salinity gradients in Shyamnagar, Bangladesh. Twenty-five roadside quadrants were systematically selected and studied for herbaceous plant diversity and soil properties. Soil Electrical conductivity (EC) and moisture show a generally rising trend from the north to the south. Moreover, the quadrants closer to the river or aquaculture with low elevation represent the salinity hot spots. About 1116 herbaceous plants belonging to 11 species were recorded. *Croton bonplandianum baill* is the dominant species and showed higher adaption capacity against soil EC values. Four out of 25 quadrants with higher EC, moisture and lower elevation recorded no plants. The herbaceous plant biodiversity reveals a strong spatial pattern and tendency to shrink with the rise of soil salinity while progressing towards the southeast direction. The area shows aggregated population, contagious distribution of plant species, and accommodates four dominant clusters. Roadside herbs not only hold medicinal value but also offer important cooking fuel choices to the fuel-scarce coastal communities. The outcome of this study provides important insights into herbaceous plant diversity and its relationships with soil salinity. Overall, the study outcomes improve our understanding of the impact of environmental stressors on the distribution of herbaceous plants in the coastal area.

## Highlights

• Explains phytosociology of the herbaceous plant community against the soil salinity.
• First report of salinity impact on herbaceous plant communities in Bangladesh.
• Soil salinity strongly affects the herbaceous plant diversity in the coastal area.
• Land elevation and soil moisture are other environmental stressors in the area.

## 1 Introduction

Salinity intrusion has been a growing problem in coastal Bangladesh, especially in low-lying areas (Dasgupta et al., 2015a). Bangladesh has been reported an increase in salinity in river water (Dasgupta et al., 2015b; Nicholls et al., 2007) and soils (Shahid 2013), particularly in coastal parts. Salinity problems in south-western coastal Bangladesh are said to be created both by the natural (i.e. seawater intrusion during cyclones, backwater effects, sea-level rise, etc.) and human systems (shrimp cultivation; lowering of freshwater inflow, etc.) (Hasan et al., 2019; Kumar et al., 2019; Anjum et al., 2018). Soil Resources Development Institute (SRDI 2010), Bangladesh dataset suggests that the salinity affected area has climbed by 1.27 times between 1973 (83.3 million ha) and 2009 (105.6 million ha).

The rise of salinity instigates several adverse effects on the ecology, economy, and society (Adger et al., 2018). Salinity creates challenging conditions for the normal crop production (Alam et al., 2017), drinking water options (Nahian et al., 2018; Rahman et al., 2017), changes in the volume and diversity of ecosystem services (Dearing and Hossain 2018), shrinkage of livelihood options, and social instability (Habiba et al., 2014). Salinity directly or indirectly mediated extensive changes in land cover and land uses in major parts of coastal Bangladesh (Hasan et al. 2019, 2020; Parvin et al., 2017). Salinity reduces soil fertility (Zhang et al., 2019), relegates germination and plant growth (Rashid et al., 2004), intensifies insect and disease infestation (Miah et al., 2004), and thus results in loss of productivity (Islam et al., 2021; Anik et al., 2018). Glycophytes like rice, wheat, and corn can not complete their life cycle under high soil NaCl concentrations (Flower et al., 2015). Chaffey et al. (1985) reported a reduction of rice production by 40% due to an increase of salinity from 20 mS cm^−1^ and 48 mS cm^−1^. As a result, local farmers opted for aquacultural practices to adjust to the changing conditions as an alternative to traditional agriculture. In Shyamnagar Upazila, aquaculture and agri-aquacultural land use have been increased by 1.17 times during the last 20 years. At the same time, agricultural land uses not only shriveled by 45% (Islam et al., 2012) but also witnessed an extinction of 19 local rice varieties (Islam et al., 2003) and a drop in crop diversity from 2.77 to 0.69 (Uddin et al., 2010) in the area.

Similar to the agricultural systems, salinity affects vegetation coverage and composition. Although the salinity-driven changes in the total area of Sundarbans are not prominent, the internal arrangement and plant species composition has been altered significantly (Rahman et al., 2011). Uddin et al. (2010) reported a decline in plant diversity both in the forests and non-forest land uses. In the forest system (Sundarbans), salinity-sensitive species (*H. fomes*) are being replaced naturally by those of salinity-tolerant (*E. agallocha*) (Rahman 2020; Zaman et al., 2013). On the other hand, in the non-forest systems, people tend to plant salinity-tolerant exotic species (*A. indica, A. lebbeck, A. nilotica*) due to their higher adaptation capacities as compared those of native ones (*A. Heterophyllus, M. indica, C. Nucifera; A. Zapota*) (Dutta and Iftekhar 2004). Several studies aimed to investigate the impact of salinity on the tree diversity of Sundarbans (Ahmed et al., 2011; Aziz and Paul 2015; Zaman et al., 2013). Despite this growing evidences about the undesirable impact of salinity on the plant communities, no studies have been conducted on the non-forest systems (Uddin et al., 2010). Local people adopted salinity-tolerant crops or plants. The plant and crop species grown in the non-forest land-use systems are principally selected by humans. Therefore, changes in trees or cultivated crops‘ biodiversity indices may not necessarily reflect the natural adaptations against the changing salinity conditions.

On the other hand, herbaceous plant accompaniments of roads (tree lines, green belt, road embankment, road-side ditches, verges, the vegetation of divider strip) grow naturally on the slopes of the road. Roadside plants have been studied for biodiversity, species richness, plant dispersal and to detect the responses to the environmental changes. Šerá (2010) studied the influence of road type on herbaceous vegetation structure. Shameem et al. (2010) reported that herbaceous species composition typically changes in space and time due to a multitude of factors, such as grazing, fire, rainfall, altitude, and soil properties (i.e. salt content). The ability to tolerate saline soil conditions is crucial for the herbaceous species growing along roads. Therefore, studies on herbaceous plant biodiversity must provide insights into the natural adjustments of plant species to salinity. Thus the present study aims to supplement data to the existing knowledge regarding the abundance and species richness of herbaceous plant population along the soil salinity gradients in the locality of Shyamnagar Upazila, Bangladesh. This study mainly achieved two objectives. Firstly, it investigates the phytosociology and diversity of roadside herbaceous plants. Secondly, it explores the soil properties that affect the spatial distribution of roadside herbaceous plants. To the best of our knowledge, this is the first report that provides insights about herbaceous plant diversity and their relationships with the salinity in a coastal area of Bangladesh.

## 2 Methods and materials

### 2.1 The study area

**Figure 1:**
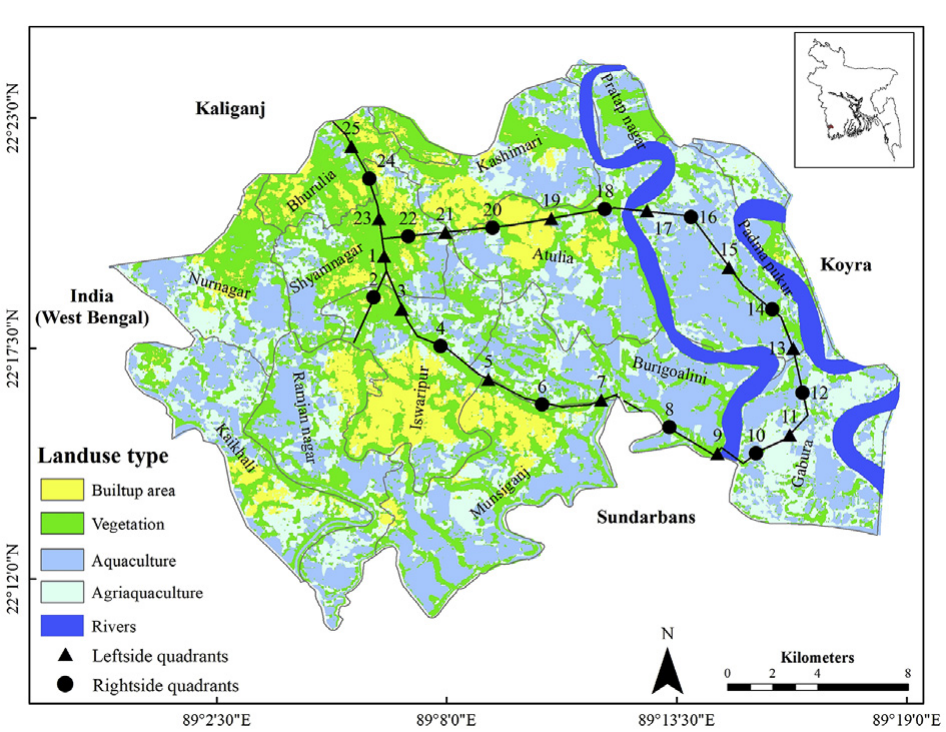
A classified map of Shyamnagar indicating land use type and sampling locations (produced from Sentinel-2; image date: 17 October 2016; accessed on 28 October 2016).

**Figure 2:**
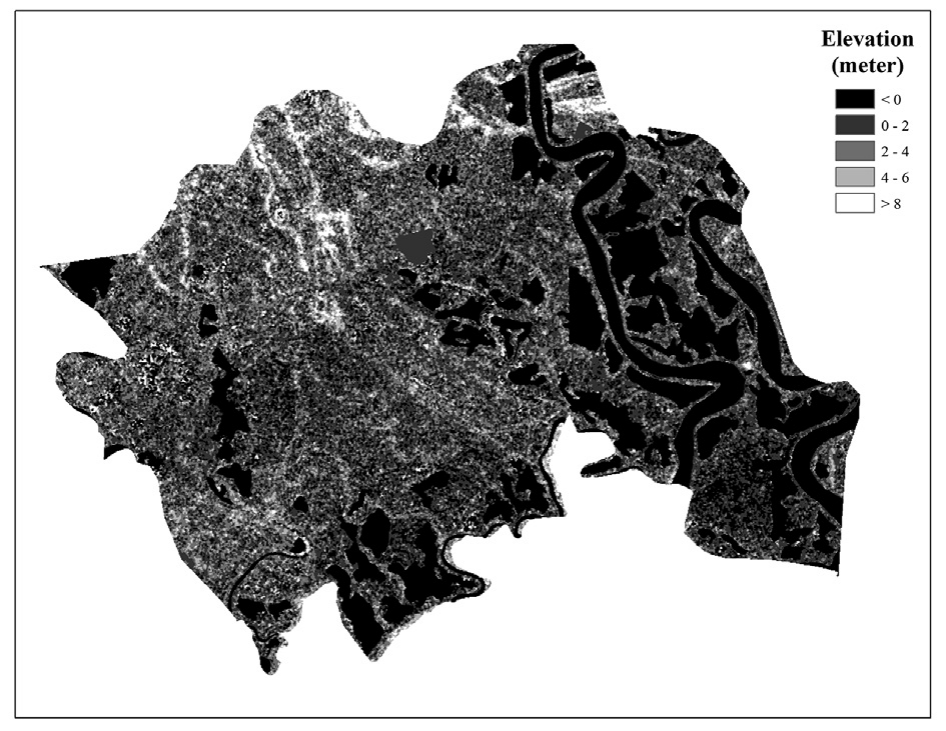
Elevation map of Shyamnagar (produced from SRTM datasets; accessed on 24 November 2017).

The study was conducted in Shyamnagar Upazila (sub-district) under Satkhira district-one of 19 coastal districts of Bangladesh. Shyamnagar (Figure 1) is the largest Upazila (an administrative unit) of Bangladesh and occupies an area of 1968.24 km^2^ including forest (82.5% and non-forest (17.5%) part). Shyamnagar Upazila consists of 13 administrative unions and accommodates more than 0.3 million people with a population density of 921 people km^−2^ in the non-forest part (national 1116 km^−2^) (Bangladesh Bureau of Statistics, 2011). The area is bounded by Sundarbans reserve forest and the Bay of Bengal to the south, West Bengal, India to the west, Koyra and Assasuni Upazilas to the east and Kaliganj, and Assasuni Upazilas to the north. Raymangal, Kalindi, Kobadak, Kholpetua, Arpangachhia, Malancha, Hariabhanga, and Chuna are major rivers of the study area. Agri-aquacultural land use (60.65%) dominates in the area followed by a built-up area (10.32%) and roads and homestead vegetations (29.03%). The Shyamnagar Upazila has a 67 km paved road, 35 km semi-paved (herringbone), and 811 km earthen road. The area is relatively flat with a maximum elevation of 13 m above mean sea level (Figure 2). The area is within 49 and 63 km of the Bay of Bengal.

### 2.2 Quadrant study

The study was carried out in 25 roadside quadrants. The size of each quadrant was 1 × 1 m in dimension as suggested by (Saxena and Singh 1982) for herbs. The sampling quadrants were selected systematically from either side of the Shyamnagar- Iswarpur – Burigoalini – Gabura – Padmapukur – Atulia – Gopalpur – Bhurulia connecting road network maintaining an approximate distance of 2 km between the quadrants. The whole length of the studied road was approximately 56 km. Sampling quadrants were targetted preliminarily with the help of the sampling function of ArcGIS software (version 10.6). In the field, the preliminary targetted sampling locations were identified with the help of a GPS machine (Garmin -GPSMAP-78S). In case, a targetted sampling quadrant was found to be influenced by the local anthropogenic activities (i.e. bridge, culvert, homestead, Bazar), the sampling quadrant was then shifted forward for another 50 m. The first quadrant was selected from the left side of the road, while the subsequent one was chosen from the right side and continued the fashion until the end. Thirteen quadrants were chosen from the left side and the 12 were from the right side. The study took the opportunity to further characterize the sampling quadrants as the ″countryside″ and ″riverside″ considering the face of the quadrant to the Kholpetua River. Since only 11 out of 25, sampling quadrants were close to the river, these 11 quadrants were analyzed while comparing ″countryside″ and ″riverside″ quadrants. Six sampling quadrants (8, 10, 11, 12, 14, 16) were termed as the riverside (chosen from the riverside of the road), while the remaining five (7, 9, 13, 15, 17) were said to be the countryside (chosen from opposite to the river). The sampling started from Shyamnagar and ended up at the farthest distance of Bhurulia. Sampling locations and quadrant IDs are shown in Figure 1. The sampling quadrants were selected, squared with a plastic measuring tape, and thereafter all herbaceous plants within the quadrants were identified individually, counted, and recorded. The study was carried out during mid-November 2016, representing the dry season only.

### 2.3 Phytosociology and biodiversity indicators

The formula and assumptions used for calculating different phytosociological and biodiversity attributes are provided in Table 1. Naturally grown vegetation in a particular area is a result of interactions among multiple edaphic, biotic and climatic factors (i.e. competition, grazing, soil type, nutrient availability, precipitation, etc.) (Dohn et al., 2013). However, this study hypothesized that soil salinity was the principal determinant of the plants' success in the area. The dominance of identified herbaceous plant species was assigned based on the estimated IVI values (please see Table 1 for IVI calculation).

**Table 1:** Formula and assumptions for different phytosociological and biodiversity attributes calculation.

Sl	Attribute	Formula or assumptions	Reference
1	% Frequency (F)	$\text{F}=\frac{Number\;of\;quadrants\;in\;which\;a\;species\;occurs}{Total\;number\;of\;quadrants\;studied}\times 100$	(Curtis and McIntosh 1950)
2	Abundance (A)	$\text{A ​}=\frac{Total\;number\;of\;individualsin\;all\;quadrants}{Number\;of\;quadrants\;in\;which\;a\;species\;occurs}$	
3	Important value index (IVI)	IVI = RF + RD + Rdom. where, $\text{RF}=\frac{Number\;of\;quadrants\;in\;which\;a\;species\;occurs}{Total\;number\;of\;all\;the\;species\;in\;the\;quadrant}\times 100$ $\text{RD}=\frac{Tot.number\;of\;individuals\;of\;a\;particular\;species\;in\;all\;quad.}{Tot.number\;of\;individuals\;of\;all\;the\;species\;in\;all\;quad.}\times 100$ $\text{RDom}.=\frac{Total\;basal\;area\;of\;a\;particular\;species\;in\;5\;quadrants}{Total\;basal\;area\;of\;all\;the\;species\;in\;5\;quadrants}\times 100$ Basal area = πr^2^; where r = radius of the stem	
4	Frequency abundance ratio (A/F)	0.025 > A/F = regular distribution; 0.025 > A/F > 0.05 = random distribution and A/F > 0.05 = contagious distribution	(Cottam and Curtis 1956)
5	Simpson diversity index (1-D)	$1-\text{D}=1-\overset{S}{\underset{i=1}{\sum }}\frac{n_{i}\left(n_{i}-1\right)}{N\left(N-1\right)}$ where; *n* = number of individuals of one particular species found; *N* = total number of individuals found	(Simpson 1949)
6	Species evenness (Eh)	$\text{Eh}=\frac{-\sum p_{i}\left(lnp_{i}\right)}{\ln\left(S\right)}$ where; *p* = *n/N; ln* is the natural log, and *S* is the number of species	(Pielou 1966)
7	Species richness (R)	$\text{R}=\frac{\text{S}-1}{\ln\left(N\right)}$	(Margalef 1958)
8	Raunkiaer species classes	Class A = (1–20) % F; Class B = (21–40) % F; Class C = (41–60) % F; Class D = (61–80) % F; Class E = (81–100) % F; where F = Frequency	(Just and Raunkiaer 1934)

### 2.4 Soil sample collection and analysis

Once all the herbaceous plants were uprooted and counted, soil samples were collected. Five soil samples (four corners and the center) from the top 15 cm were collected, mixed, and carried in a pre-labeled ziplock plastic bag. Sampling locations and elevation data of each sample were recorded using the GPS machine. At the laboratory, soil samples were air-dried and screened through a 0.5 mm sieve. The sieved soil samples were then used to prepare a 1:5 soil: distilled water suspension stock solution. Electrical conductivity (EC) and pH were then measured using pre-calibrated handheld portable meters (Hanna HI-99301and Hanna HI-8424). EC values were interpolated following the IDW technique using the ArcGIS (10.5) software package. Gravimetric soil water content (%) was estimated using Eq. (1).

(1) $$\text{Soil}\;\text{water}\;\text{content}\left(\text{\%}\right)=\frac{\text{mass}\;\text{of}\;\text{moist}\;\text{soil}\left(\text{g}\right)-\text{mass}\;\text{of}\;\text{dried}\left(\text{oven}\right)\;\text{soil}\;\left(\text{g}\right)}{\text{mass}\;\text{of}\;\text{dried}\left(\text{oven}\right)\;\text{soil}\;\left(\text{g}\right)}\times 100$$

### 2.5 Normalized Difference Vegetation Index (NDVI) calculation

Normalized Difference Vegetation Index (NDVI) is a popular tool to characterize the greenness of a land-use with the help of remote sensing imageries. NDVI ranged from -1 to 1. Negative values of NDVI correspond to water. Values close to zero (-0.1 to 0.1) generally correspond to barren areas of rock, sand, or snow. Positive values (approaching 1) indicate the presence of healthy vegetation (chlorophyll). To validate the study outcome, NDVI was calculated from the Landsat-8 image taken on 20 October 2016 (downloaded freely from https://glovis.usgs.gov/ on 11 November 2016). Radiometric and atmospheric correction was performed using the ERDAS Imagine (version 2015) software according to the protocol provided by the image source. The NDVI was calculated following Eq. (2). The NDVI raster values corresponding to the soil sampling locations were then extracted using ArcGIS (version 10.6) software.

(2) $$\text{NDVI}=\frac{\text{NIR}-\text{R}}{NIR+R}$$

where,

*NDVI* = Normalized Difference Vegetation Index; R and NIR are the Red and Near-infrared reflectance.

### 2.6 Statistical analysis

Statistical analysis was performed using MS Excel and IBM SPSS Statistics for Windows, Version 16.0 (SPSS Inc., Chicago, IL, USA). Pearson correlation and two-tailed student's t-test was performed to verify the statistical significance at 95 and 99% confidence level. The single link dendrogram based on euclidean distance (z-scores standardized) was plotted to understand the hierarchical clustering of studied quadrants.

## 3 Results

### 3.1 Elevation and soil electrical conductivity

Elevation data suggests that the studied quadrants have a mean elevation of 4.24 m (maximum 9.0 m; minimum 1.0 m; median 4.0 m) above mean sea level (msl) with a clear slope from north to south. Quadrants located at the south-western end (Gabura and Burigoalini union) show the lowest elevation and tend to rise while approaching either towards Shyamnagar via Iswaripur or Bhurulia via Padmapukur and Atulia. The elevation of the study area is shown in Figure 2. The soil properties of the studied quadrants are given in Table 2.

**Table 2:** Location, soil properties, and biodiversity indicators of studied quadrants.

Quad. ID	Quad. face	Quad. location		Elev. (m, a.s.l.)	Soil properties				NDVI	Biodiversity indicators		
		Long.	Lat.		EC (*mS/cm*)	pH	Moisture (%)	Textural class∗		Simpson index (*1-D*)	Species richness (R)	Species evenness (*Eh*)
1	L	89.11	22.33	6.0	0.24	6.31	4.30	Silty clay loam	0.65	0.66	0.49	0.97
2	R	89.10	22.31	4.0	0.66	6.61	6.87		0.58	0.58	0.54	0.86
3	L	89.15	22.28	6.0	1.61	7.65	5.64		0.48	0.19	0.22	0.49
4	R	89.20	22.27	3.0	7.49	5.49	6.75		0.16	0.36	0.43	0.72
5	L	89.20	22.27	5.0	1.01	7.25	7.34		0.46	0.00	0.00	0.00
6	R	89.24	22.25	4.0	1.20	7.35	10.10		0.51	0.64	0.83	0.81
7	L; C	89.24	22.25	5.0	1.68	6.82	13.23		0.30	0.59	0.65	0.87
**8**	**R; Riv**	**89.25**	**22.25**	**1.0**	**2.74**	**7.22**	**21.12**		**-0.03**	**0.00**	**0.00**	**0.00**
**9**	**L; C**	**89.25**	**22.25**	**2.0**	**4.62**	**6.95**	**15.74**		**-0.17**	**0.00**	**0.00**	**0.00**
**10**	**R; Riv**	**89.26**	**22.25**	**4.0**	**9.24**	**7.20**	**17.31**		**-0.18**	**0.00**	**0.00**	**0.00**
**11**	**L; Riv**	**89.27**	**22.26**	**3.0**	**12.60**	**7.60**	**16.76**		**-0.01**	**0.00**	**0.00**	**0.00**
12	R; Riv	89.28	22.27	2.0	2.08	7.45	11.75		0.02	0.00	0.00	0.00
13	L; C	89.27	22.29	4.0	1.82	7.57	12.24		0.09	0.67	0.83	0.85
14	R; Riv	89.27	22.30	6.0	0.78	7.55	11.55		0.51	0.36	0.43	0.72
15	L; C	89.25	22.32	4.0	2.68	7.70	11.87		-0.02	0.47	0.33	0.92
16	R; Riv	89.23	22.34	2.0	6.27	5.71	9.32		-0.03	0.41	0.48	0.65
17	L; C	89.20	22.35	5.0	1.40	6.70	8.73		0.43	0.43	0.62	0.61
18	R	89.19	22.35	3.0	5.54	7.10	9.74		-0.02	0.57	0.58	0.83
19	L	89.17	22.34	4.0	2.69	7.07	7.63		0.00	0.36	0.44	0.55
20	R	89.16	22.34	5.0	2.71	6.75	5.43		-0.01	0.49	0.62	0.66
21	L	89.12	22.34	3.0	5.19	7.10	4.67		-0.01	0.42	0.24	0.87
22	R	89.10	22.34	9.0	0.41	7.46	5.55		0.61	0.59	0.55	0.86
23	L	89.11	22.34	7.0	0.67	7.51	7.32		0.48	0.63	0.61	0.92
24	R	89.10	22.35	4.0	1.35	6.93	4.53		0.44	0.74	0.84	0.81
25	L	89.10	22.35	5.0	1.14	7.10	5.34		0.59	0.62	0.68	0.82

L = leftside; R = rightside; Riv = riverside; C = countryside; EC = Electrical conductivity; m, a.s.l. = meter above sea level; NDVI = Normalized Difference Vegetation Index;

∗ Adapted from SRDI soil series information.

Soil electrical conductivity (EC) among the studied quadrants ranges from 0.24 and 12.6 mS/cm (Table 2) with a distinct spatial pattern. Soil EC shows a significant negative correlation with quadrants elevation at 99% confidence level (*r = -0.51; df =23; p < 0.00*) and an increasing trend from the north-west to the south-east.

### 3.2 Soil pH and moisture contents

Soil pH (range between 5.49 and 7.70) indicates that the soils are saline as suggested by (Brady and Weil 2004). The maximum pH was recorded at quadrant # 15 at Padma Pukur Union and the lowest was at quadrant # 4 at Iswarpur Union. Soil moisture contents among the studied quadrants range from 4.3 and 21.12 %. As illustrated in Table 3, soil moisture shows significant negative correlation with quadrants elevation at 99% confidence level (*r = -0.52; df = 23; p < 0.00*) and significant positive correlation with soil EC values at 95% confidence level (*r = 0.45; df = 23; p < 0.05*).

**Table 3:** Pearson correlation coefficient (*r*) of soil parameters, biodiversity index, and vegetation coverage.

	Soil EC	Soil pH	Soil moisture	Quadrant elevation	Simpson index (1-D)	Species evenness (Eh)	Species richness (R)	NDVI
Soil EC	1							
Soil pH	-0.173	1						
Soil moisture	.448∗	0.271	1					
Quadrant elevation	-.509∗∗	0.253	-.522∗∗	1				
Simpson index (1-D)	-.486∗	-0.168	-.597∗∗	.447∗	1			
Species evenness (Eh)	-.443∗	-0.186	-.632∗∗	.471∗	.945∗∗	1		
Species richness (R)	-.463∗	-0.186	-.515∗∗	0.388	.943∗∗	.839∗∗	1	
NDVI	-.682∗∗	0.032	-.572∗∗	.717∗∗	.506∗∗	.474∗	.473∗	1

∗ Correlation is significant at the 0.05 level (2-tailed).

∗∗ Correlation is significant at the 0.01 level (2-tailed).

### 3.3 Phytosociology

A total of 1116 herbaceous plants belonging to 11 species were recorded. *Croton bonplandianum baill* recorded the highest frequency (84%) and abundance (30.33) among the species. The frequencies of plant follow the order: *C. bonplandianum baill* (84%) > *A. lividus* (52%) > *A. dracunculus* (24%) > *P. hysterophorus* (20%) > *A. retroflexus* (20%) > *I. verticillatum* (12%) > *O. vulgare; H. Indicum; R. tuberosa* (8%) > *P. acinosa; A. aspera* (4%). *Croton bonplandianum baill* was the dominant species in 13 quadrants with an overall IVI of 114.47. Besides, *Parthenium hysterophorus* (IVI 31.58) and *Origanum vulgare* (IVI 17.79) species dominated in two quadrants. The overall rank order of the species could be written as *C. bonplandianum baill* > *A. lividus > P. hysterophorus > I. verticillatum > A. retroflexus > A. dracunculus > O. vulgare > H. indicum > R. tuberosa > P. acinosa > A. aspera.*

**Figure 3:**
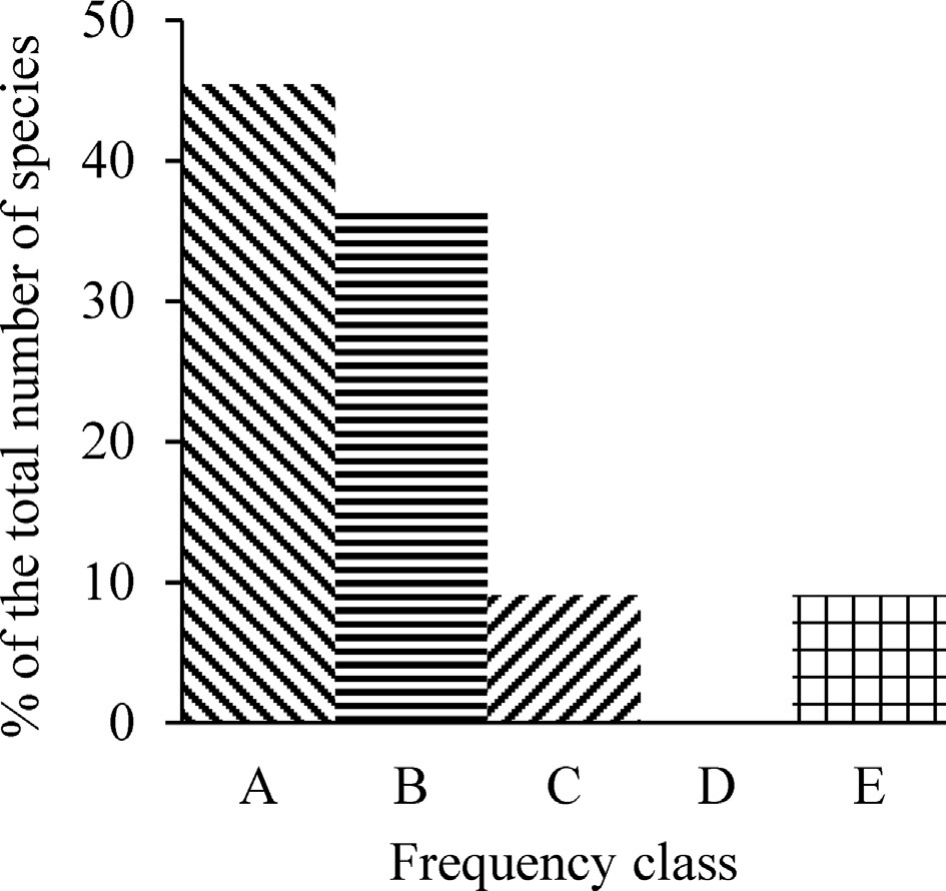
Histogram of vegetation class standard frequency diagram.

The phytosociological information of the studied quadrants is provided in Table 4. (Just and Raunkiaer 1934)'s standard frequency diagram (Figure 3) suggests a uniform distribution of species along with the studied quadrants.

**Table 4:** Phytosociological information of the studied quadrants.

Sl #	Species	Local name	Number of quadrants recorded	Total individual recorded	Frequency (%)	Abundance	A/F	IVI	Aggregation
1	*Croton bonplandianum baill*	Bondhone/ Bon tulsi	21	637	84	30.33	0.36	114.47	Aggregated
2	*Amaranthus lividus*	Bounoti shak	13	184	52	14.15	0.27	48.51	Aggregated
3	*Parthenium hysterophorus*	Meye ful	5	97	20	19.40	0.97	31.58	Aggregated
4	*Amaranthus retroflexus*	Shishanondo	5	46	20	9.20	0.46	19.28	Aggregated
5	*Illecebrum verticillatum*	Kata ful	3	43	12	14.33	1.19	19.62	Aggregated
6	*Artemisia dracunculus*	Udd gach	6	43	24	7.17	0.30	19.11	Random
7	*Origanum vulgare*	Jauna	2	31	8	15.50	1.94	17.79	Random
8	*Heliotropium indicum*	Hatishura	2	18	8	9.00	1.13	11.71	Aggregated
9	*Ruellia tuberosa*	Potpoti	2	8	8	4.00	0.50	7.02	Random
10	*Phytolacca acinosa*	Arol gach	1	5	4	5.00	1.25	5.87	Random
11	*Achyranthes aspera*	Apang	1	4	4	4.00	1.00	5.03	Random

### 3.4 Biodiversity indicators

**Figure 4:**
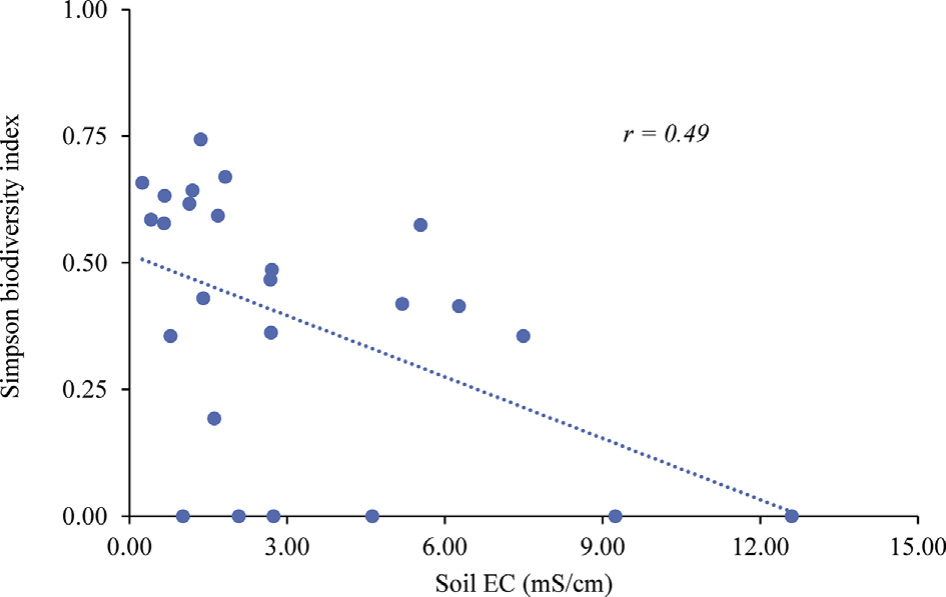
Simpson biodiversity index versus soil salinity.

Simpson biodiversity index value ranged between 0 and 0.74 (mean 0.39, median 0.43). Quadrants chosen from the left side of the road (mean 0.39) do not show significant differences with those from the right side (mean 0.38) in terms of Simpson index value at 95% confidence level (*t-test; p = 0.91; df = 21*). In contrast, the countryside (mean 0.43) quadrants display wider biodiversity as compared to those of riverside (mean 0.13) with no statistical significance (*t-test; p = 0.09; df = 7*). Overall, the herbaceous plant biodiversity portrays statistically significant negative correlation with the soil EC at 95% confidence level (*r = -0.49;df = 23; p < 0.05*) (Figure 4).

Biodiversity datasets suggest a general declining trend with the increase of soil salinity. Moreover, biodiversity value exposes statistically significant positive correlation with the quadrant elevation at 95% confidence level (*r = -0.45; df =23; p < 0.05*). Furthermore, herbaceous plant biodiversity reveals a strong spatial pattern. Plant biodiversity tends to shrink towards the southeast direction.

Species evenness varied between 0 and 0.97 (mean 0.59, median 0.72). Maximum evenness was recorded at quadrant IDs 1, 15 and 23. Species richness ranged between 0 and 0.84 (mean 0.42, median 0.48). Maximum species richness was recorded at quadrant IDs 24, 13 and 6. Species richness shows significant negative correlation with soil EC at 95% confidence level (*r = -0.46;df = 23; p < 0.05*) and soil moisture at 99% confidence level (*r = -0.51; df = 23; p < 0.00*).

### 3.5 Cluster analysis

**Figure 5:**
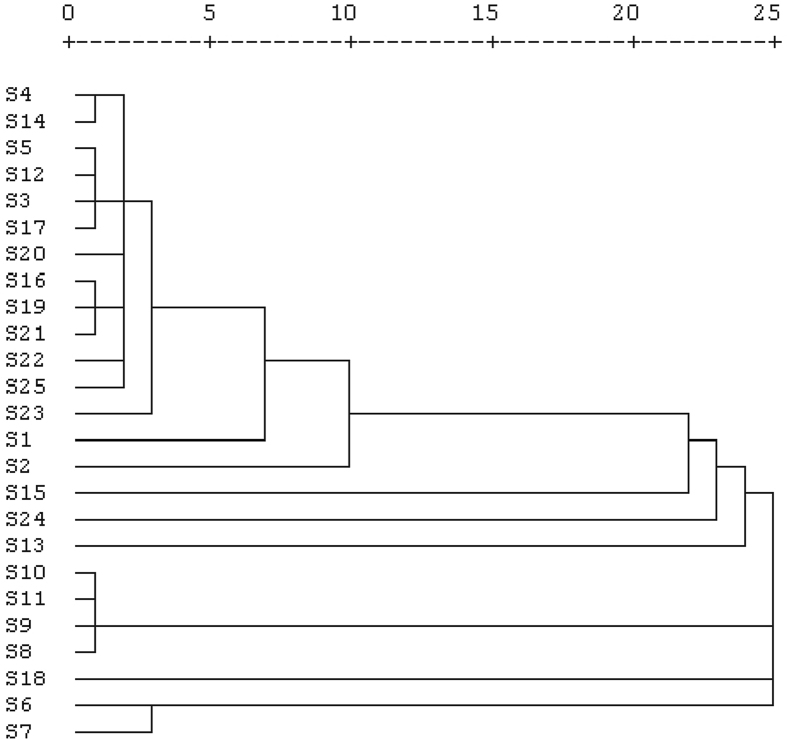
Single linkage dendrogram of studied quadrants based on species abundance.

Cluster analysis suggests that the studied quadrants fall into four prominent clusters (Figure 5). Studied quadrants 8, 9, 10, and 11 represent clusters with no plants. Quadrants 13, 15, and 24 are characterized by high soil moisture and least salinity. *Parthenium hysterophorus* is the representative plant of this cluster. Quadrants 6, 7, and 18 showed the outlier characteristics. The rest of the quadrants are mixed in nature and dominated by *Croton bonplandianum baill* and *Amaranthus lividus*.

## 4 Discussion

### 4.1 Soil properties

Estimated soil EC and moisture values showed good agreement with Shaibur et al. (2017) and Kumar et al. (2019). In a study on the agricultural soil properties at Gabura and Burigoalini union (closer area of quadrant # 5 to 12 of this study), Shaibur et al. (2017) reported slightly higher soil EC values. Shaibur et al. (2017) conducted their study under drier conditions (March) as compared to that of the present study (November). Moreover, agricultural soils are more likely to be exposed to salt deposition due to its more susceptibility to inundation and thus could show slightly high EC values. In another study, Kumar et al. (2019) reported a dry-season soil salinity range between 3.92-7.69 mS cm^−1^ in Shyamnagar soils.

The increasing trend of soil EC values identified in the interpolated soil EC map (Figure 3) is also consistent with the conclusions made by Siddiqi (2001). Siddiqi (2001) concluded that the salinity of the Sundarbans and its adjacent coastal areas increases from the north to the south. Relatively higher soil moisture and salinity in the southern quadrants were determined mainly by two factors. Firstly, these quadrants are relatively lower in elevation, and thus subject to frequent saline water inundation during cyclones and floods. Secondly, the proximity of these quadrants to the river (Chuna River, Kholpetua River, and Kobadak River) or aquaculture hotspots might have influences on the soil salinity. Lower elevation, high water table, and the effect of capillary rise have a combined effect on the salinity levels.

### 4.2 Phytosociology

The absence of any plant species in the studied quadrants might be associated with high soil salinity (mean EC = 7.3 mS cm^−1^; all quadrants mean 3.11 mS cm^−1^), lower elevation (mean 2.5 m; all quadrants mean 4.24 m), and high soil moisture (17.7%; all quadrants mean 9.63%). Soil salinity shows significant correlations with both quadrant elevation and soil moisture content (Table 3). Low elevation allows the quadrants to go under saline water easily. Moreover, high soil moisture implies the presence of saline water inside the soil pores. Therefore, the prevalence of low elevation and high soil moisture create a high saline condition for the plants to be survived. High salinity has two common effects on the plants. Firstly, salty induces osmotic stress to the plants and, thus reduces their ability to uptake water. Water shortage halts the growth of root and shoots cells (Munns et al., 2000; Fricke et al., 2004). Secondly, salt creates ion toxicity to the plants. Excessive Na^+^ can harm plat metabolism and inhibits enzymes activity (Cheeseman 2013).

Among the identified herbaceous plant species, *C. bonplandianum baill* shows higher adaption capacity against soil salinity through its wider presence in quadrants with soil EC values as high as 7.49 mS cm^−1^. Two out of four quadrants where no plants were recorded have soil EC values of 4.62 mS cm^−1^ and 2.74 mS cm^−1^. However, these two quadrants show the least elevation and highest soil moisture contents as compared to those of others. Therefore, it is intuitive that soil moisture content could be the limiting factor for the growth of roadside herbs. In support (Rahman and Akter 2013), narrated that *C. bonplandianum baill* prefers dry and sandy exposed soils.

The distribution pattern of the plant species in the studied quadrants suggests that only 8.14% were randomly distributed, while the rest are aggregated. Aggregated populations indicate that the individuals of the species were found in clump. Similarly, the abundance frequency ratio of each species (>0.05) implies the species can be considered as contagious distributed and tendency to show clustering.

### 4.3 Biodiversity indicators

An insignificant difference in biodiversity indices between the right and left side quadrants indicate that either side of the road offers similar growing conditions. However, the soils of the riverside quadrants contain a higher amount of salts, therefore, it is evident that herbaceous plant biodiversity also declined. Similar findings are also widely reported for plant communities elsewhere (Dohn et al., 2013; Greenberg et al., 2006; Mitsch and Gosselink 2000).

**Figure 6:**
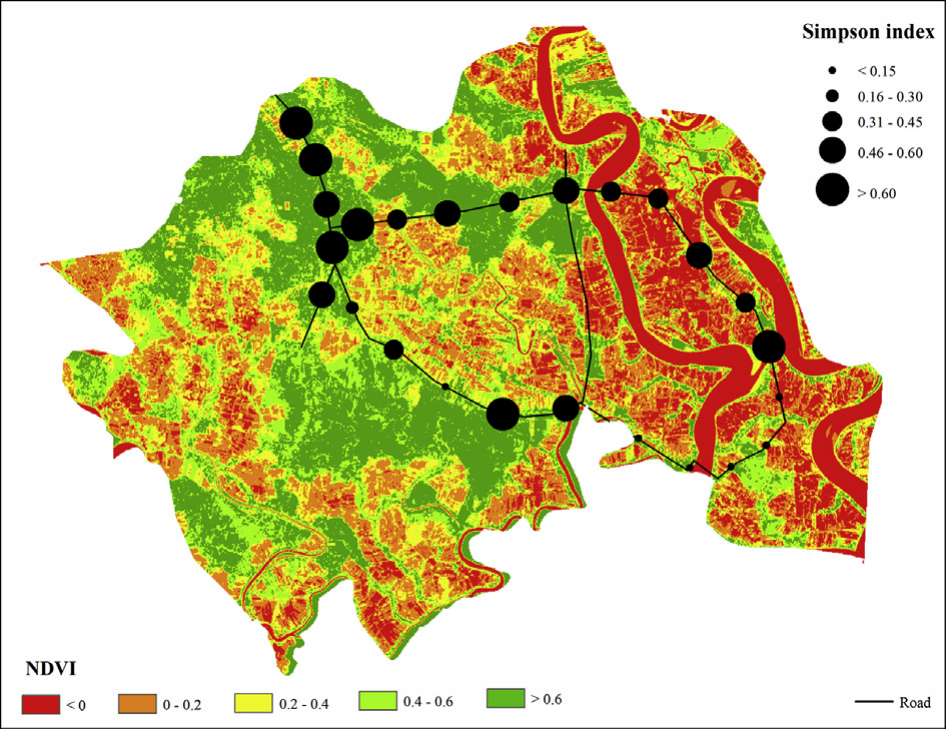
NDVI and Simpson index map.

The study outcome was also validated against the satellite image-based NDVI values. Overall the NDVI analysis indicates that the non-forest part of Shyamnagar Upazila has limited vegetation coverage. The area has around 19% waterbody (0 < NDVI), 23% bare soil or non-cultivated agricultural field (0.2 < NDVI >0), 17% grassland or cultivated agricultural field (0.4 < NDVI >0.2) and about 41% vegetation coverage (NDVI >0.4). The vegetation coverage is mainly shown along the roadside, embankments, and homesteads (Figure 6). Extracted NDVI values of the soil sampling point location represent the strong agreement (*r*^*2*^ *= 0.63*) with those of Simpson index values.

Cluster analysis implies that quadrants 13, 15, and 24 have strong similarities, and thus quadrants 24 and 13 recorded close richness values. On the other hand, 18 out of 31 *Origanum vulgare* were recorded in quadrant # 6. Quadrant # 6 was relatively more exposed to sunlight as compared to those of higher elevation and lesser soil EC quadrants (1–5; 20–25). Exposure to sunlight could favor the plants to be flourished. On the other hand, quadrant # 13, seems to have anthropogenic influences. It was close to a local Bazar and widened with fresh soil (approximately 1.5 years before the study period). Such external influence might support higher species richness and thus produce different outcomes as compared to those of adjacent quadrants (12, 14).

## 5 Conclusions

This study assessed the spatial distribution of roadside herbs in a highly salinity affected and the Sundarbans adjacent non-forest coastal sub-district (Shyamnagar) of Bangladesh. The outcome of this study answered at least three research questions. Firstly, it investigated the soil salinity gradient along with the roadside soils of the area. It suggests that soil salinity tends to rise towards the southeast direction and discloses salinity hotspots nearer to the river or aquacultural land uses. Secondly, it verifies the impacts of soil salinity on the roadside (naturally grown) herbaceous plant diversity. The study outcome reveals an inverse relationship between soil salinity and biodiversity indices. Finally, it identifies the plant species that showed the best adaptions under salinity conditions. The results were validated against available literature and independent datasets. Roadside herbs could offer important ecosystem services for the salinity-affected aquaculture intensified Shyamnagar area. The local community has been facing a fuelwood and fodder crisis due to the decline in agricultural residues (agricultural residues are traditionally used for cooking). Therefore, people often tend to depend on the Sundarbans for their fuelwood requirements. Roadside herbs could add supplemental support in fuelwood sources under a resource-scarce situation. Moreover, herbs could aid traditional health care efforts with their medicinal values. Some areas of Shyamnagar are very remote and hard to reach, and thus people rely on traditional herbal medicines. Further study should focus on the role of herbaceous plants on the local micro-ecosystems, use values (fuelwood, fodder, and others), and medicinal importance. Therefore, this study provides baseline information to the researcher and policymakers about the changes of the herbaceous plant community over space and salinity gradient. The outcome of this study could also contribute to a growing sphere of ecological research in the sense of understanding the impact of environmental stressors (i.e. salinity, temperature, soil moisture, and elevation) on the distribution of plant species along spatial gradients.

## Declarations

### Author contribution statement

Abu Selim, Ehsanul Bari: Conceived and designed the experiments; Performed the experiments; Analyzed and interpreted the data.

Md. Hasibur Rahaman, Mohammad Mahfuzur Rahman: Conceived and designed the experiments; Contributed reagents, materials, analysis tools or data; Wrote the paper.

### Funding statement

This research did not receive any specific grant from funding agencies in the public, commercial, or not-for-profit sectors.

### Data availability statement

Data included in article/supplementary material/referenced in article.

### Declaration of interests statement

The authors declare no conflict of interest.

### Additional information

No additional information is available for this paper.
